# Epidemiology and management of bacterial vaginosis in Dakar, Senegal

**DOI:** 10.1186/s12905-026-04443-w

**Published:** 2026-04-09

**Authors:** Cheikh A.T. Cissé, Mouhamadou M. Niang, Julie Escola, Barbara Quioc-Salomon, Charlotte P.J. Talbot, Frédéric Carrois

**Affiliations:** 1 Gynecology-Obstetric Department, Institut d’Hygiène Social (IHS), Dakar, Sénégal; 2 Innotech International, 22 avenue Aristide Briand, Arcueil, 94111 France

**Keywords:** Vulvovaginitis, Vaginal Infections, Vulvovaginal Infections, Bacterial Vaginosis, Neomycin, Nystatin, Polymyxin B.

## Abstract

**Background:**

In Africa, vulvovaginal infections represent a public health issue due to their frequency and complications. Recent studies have shown a significant shift in bacterial ecology, which is increasingly dominated by bacterial vaginosis. The PETAL study aimed to determine the epidemiological, clinical, and microbiological profile of vulvovaginal infections in Dakar (Senegal), to assess the predictive etiological value of the syndromic approach by comparing it with microbiological results, and to evaluate the clinical and microbiological efficacy and the tolerance of treating bacterial vaginosis with the Neomycin-Polymyxin B-Nystatin (NNP) combination (Polygynax^®^).

**Methods:**

This monocentric, prospective, single-arm and observational study was conducted at the Gynecology-Obstetrics Department of Institut d’Hygiène Social Hospital in Dakar. Women with suspected vulvovaginal infection underwent a clinical exam and vaginal bacteriological sampling. Based on the WHO syndromic approach, an initial diagnosis was made, and patients received NNP treatment (one vaginal capsule daily for 12 days). Clinical and microbiological outcomes were assessed on Day 15 after treatment. Consistency between the syndromic and microbiological diagnoses was evaluated using Cohen’s Kappa test.

**Results:**

Women included in this study were on average 32 years old, mostly married (87.0%) and unemployed (52.2%). Among the 224 patients analyzed, 78.6% had positive bacteriological results at Day 0. Bacterial vaginosis was confirmed in 68.7% of cases, either alone (44.6%) or associated with candidiasis (22.8%) or trichomoniasis (1.3%). Isolated candidiasis accounted for 9.8%. High mixed-infection rates highlight diagnostic challenges. The syndromic approach showed strong consistency with microbiological diagnosis for bacterial vaginosis (sensitivity: 100%; specificity: 96.1%), but lower agreement for candidiasis (sensitivity: 100%; specificity: 78%) and mixed infections (sensitivity: 59.3%; specificity: 100%). Clinical remission was achieved in 91.4% of cases and bacteriological cure in 73.3% of vaginosis cases. The treatment was well tolerated (98.5%).

**Conclusions:**

Vulvovaginal infections in Dakar are largely dominated by bacterial vaginosis. In cases of isolated vaginosis, the syndromic approach showed very good consistency with the bacteriological diagnosis, while it seems less suitable for identifying mixed infections, which account for one-third of cases. In this context, the NNP combination could be an appropriate, effective, and well-tolerated treatment for the management of bacterial vaginosis.

**Supplementary Information:**

The online version contains supplementary material available at 10.1186/s12905-026-04443-w.

## Background

The etiology of vulvovaginal infections is typically linked to an imbalance in the vaginal microbiota, characterized by an overgrowth of pathogenic microorganisms. The most commonly observed conditions include bacterial vaginosis, aerobic vaginitis, and vulvovaginal candidiasis [1–3]. Several studies published over the past 15 years have reported that the most common clinical forms of vulvovaginal infections in African countries are bacterial vaginosis and vaginal candidiasis [4–8].

Vulvovaginal infections are among the most frequent reasons for gynecological consultations, accounting for approximately 20% of cases [9, 10]. They represent a significant public health issue due to their high prevalence and the complications they may cause, particularly obstetric and oncologic risks, as well as long-term sequelae such as chronic pelvic pain and infertility [10]. They negatively impact women’s quality of life, leading to frustration, anxiety, sexual dysfunction, and persistent vulvovaginal discomfort.

Establishing the etiological diagnosis of vulvovaginal infections is often challenging, especially in low-resource settings. The World Health Organization (WHO) syndromic approach provides a valuable approach in order to establish a first diagnosis and treatment, particularly in setting where laboratory diagnosis is not possible [11]. However, a better understanding of the evolving microbiological profile of pathogens responsible for vulvovaginal infections is essential to improve the accuracy and the effectiveness of this method.

Management of vulvovaginal infections depends on the causative microorganism, with targeted antimicrobial therapy playing a key role in ensuring both clinical and microbiological improvement. Nitro-5-imidazoles remains the standard of care to date for the treatment of bacterial vaginosis, either administered orally or intravaginally [11–17]. A Cochrane meta-analysis estimated the efficacy of these treatments at approximately 80% [18], although other studies have reported cure rates as low as 60% [19]. This reduced efficacy may be partly due to the polymicrobial nature of bacterial vaginosis, typically involving *Gardnerella* spp., often in association with *Atopobium vaginae* and/or *Mobiluncus curtisii*, which exhibit differential susceptibility to metronidazole [20]. In addition, oral metronidazole is often poorly tolerated due to gastrointestinal side effects, may disturb the Döderlein flora, and can promote secondary vulvovaginal candidiasis. These limitations, combined with the risk of misdiagnosis leading to inappropriate treatment, highlight the need to evaluate alternative therapies in real-life settings. In this context, the need for alternative therapeutic options have been questioned [13, 15, 19, 21], and the in vivo efficacy of the Neomycin-Polymyxin B-Nystatin (NNP) combination as treatment for bacterial vaginosis was evaluated in the present study. In line with these diagnostic uncertainties, a community practice study reported that 42% of women presenting with vulvovaginal infection symptoms received inappropriate treatment [22], reflecting the challenges in precisely identifying the underlying etiology of infection, leading to high rates of therapeutic failure and recurrence [23]. These frequent diagnostics uncertainties, related to the diversity of pathogens, highlight the need to consider broad-spectrum therapy such as the NNP combination. This treatment, combining both antibiotic and antifungal agents, has been used for decades and shows high efficacy and good safety profile in the management of vulvovaginal infection [24].

However, clinical efficacy data on NNP combination remain limited in patients with bacterial vaginosis, particularly in African settings. A recent study evaluated the in vitro bactericidal activity of the NNP combination compared to the reference antibiotic metronidazole against the main pathogens involved in bacterial vaginosis, including *Gardnerella vaginalis*, *Atopobium vaginae*, *Prevotella bivia*, and *Mobiluncus curtisii* [17]. The findings from this study demonstrated that the NNP combination exhibited a rapid and locally effective bactericidal effect against a broad spectrum of Gram-positive and Gram-negative, and aerobic and anaerobic bacteria in comparison with the metronidazole and clindamycin.

The aim of the PETAL study (Polygynax Evaluation du Traitement en vie réelle au SénégAL) was primarily to investigate the clinical and microbiological efficacy and tolerance of the NNP combination in the treatment of bacterial, offering insights into alternative therapeutic strategies. In addition, the study sought to provide a comprehensive understanding of vulvovaginal infections in Dakar by characterizing their epidemiological, clinical, and microbiological aspects. Finally, as a secondary objective, it aimed to evaluate the relevance and diagnostic accuracy of the WHO syndromic approach by comparing clinically suspected infections with microbiological results.

## Methods

### Study design

This monocentric, prospective, single-arm and observational study was conducted in real-life conditions at the Gynecology-Obstetrics Department of Institut d’Hygiène Sociale Hospital in Dakar, Senegal, from November 1, 2023, to February 28, 2025. The NNP treatment decisions were made independently of study participation and reflected routine clinical practice. No additional procedures beyond standard care were performed for research purposes.

### Study participants

Eligible women were adults of reproductive age who presented with symptoms suggestive of vulvovaginal infection and had a current prescription for NNP treatment as part of routine clinical management. The purpose of the study was explained to eligible patients, and only participants who provided written informed consent were included in the study.

The main exclusion criteria were pregnant women, women with negative bacteriological test results, and those diagnosed with a sexually transmitted infection (trichomoniasis, chlamydia, or gonorrhea), as the empirical treatment used was not indicated for these conditions.

### Study protocol

During the inclusion visit (defined as Day 0), patients were assessed clinically (patient-reported symptoms and gynecological examination), and the WHO syndromic approach was used to establish a preliminary diagnosis of the type of vulvovaginal infection. Following the clinical exam, a vaginal swab was collected and analyzed in the laboratory to assess the following parameters: type of Döderlein flora, potassium hydroxide (KOH) test, vaginal pH, Nugent score [25], Amsel criteria [26], and microbial culture for pathogen identification.

Following sample collection, patients received empirical treatment consisting of one capsule of NNP, (Polygynax^®^ vaginal capsule, Laboratoire Innotech Internationnal) per day for 12 consecutive days. After microbial culture (6–10 days), results were compared with the initial syndromic diagnosis to adjust treatment if necessary.

On Day 15, clinical examination was performed for all patients. For patients with microbiological diagnosis of bacterial vaginosis, a second microbiological evaluation was performed on Day 15.

### Evaluation criteria

The clinical remission rate after NNP treatment was defined as the resolution of initial symptoms and assessed by the investigator following gynecological examination and patient interview at Day 15. Microbiological cure rate evaluated by comparing microbiological tests at Day 0 and Day 15. It was assessed using the following criteria: absence of pathogens on direct examination and culture, a Nugent score ≤ 6, and < 3 positive Amsel criteria. As predefined in the protocol, a vaginal sample was collected for microbiological evaluation at Day 15, including culture, Nugent score assessment, and Amsel criteria. In cases of discordance between these parameters, culture results were used to establish the final microbiological classification.

Secondary outcomes included the occurrence of adverse effects and patient satisfaction with treatment, assessed using a 5-point scale ranging from “very poor” to “very good”.

### Sample size calculation

Based on an expected clinical efficacy rate of 75% for the NNP combination, derived from previously published clinical data from the PRISM study [24], a minimum of 80 evaluable patients was required to estimate this proportion with a ± 10% margin of error and a 95% confidence interval, assuming 90% statistical power and using the Wilson score method. This threshold was defined a priori in the study protocol as a conservative and pragmatic estimate for a real-life, non-comparative study. To account for potential losses (e.g., treatment non-adherence, loss to follow-up, or incomplete data), the sample size was increased to 100 patients with confirmed bacterial vaginosis. Given that bacterial vaginosis is estimated to represent 25–50% of all vulvovaginal infections, approximately 400 women with suspected infection needed to be screened to reach the target sample. The recruitment was planned to be stopped if more than 100 patients with confirmed diagnosis of bacterial vaginosis were reached.

### Statistical analysis

Data were analyzed using univariate analysis and are presented as percentage and mean values ± standard deviation (SD) if applicable.

A Bivariate analysis was performed to identify factors associated with the occurrence of infection and those influencing treatment outcomes among patients with bacterial vaginosis.

To assess the concordance between the syndromic approach and the bacteriological analysis of vaginal samples, statistical tests used included proportion comparisons, the Chi-square (χ²) test (statistically significant link if *p* < 0.05), sensitivity and specificity measurements, positive predictive value and negative predictive value measurement, the Matthews correlation coefficient (≥ 1 = perfect prediction, < 0.6 = random prediction) and the Kappa coefficient for agreement assessment (0 ≤ K < 0.20: weak agreement, 0.21 ≤ K < 0.40: moderate agreement, 0.41 ≤ K < 0.60: substantial agreement, 0.61 ≤ K < 0.80: strong agreement and 0.81 ≤ K ≤ 1: almost perfect agreement).

Statistical analysis was performed using KoboCollect (Version V 2024.2.4, USA). Cohen’s Kappa coefficient was calculated using the Cohen’s Kappa Calculator, while sensitivity, specificity, predictive values, and Matthews correlation coefficient were computed using the Confusion Matrix tool available at https://www.dcode.fr/matrice-de-confusion. These tools were selected for their transparency and reproducibility, allowing direct input of binary classification data. A *p* ≤ 0.05 was considered significant and is denoted with one asterisk.

According to rounding conventions, the results may therefore exceed or fall below 100% (when a decimal value is exactly 0.5, the number is rounded up to the next integer).

### Ethics approval and consent to participate

Participation in the study was entirely voluntary. Each patient received counseling during which the study objectives and procedures were fully explained. Participants were ensured full understanding of the information provided, emphasizing their right to withdraw at any time without consequence. Patients were reassured that refusal to participate or withdrawal of consent during the study would in no way affect the quality of care they received. This monocentric, prospective, single-arm and observational study was conducted in real-life conditions without any additional procedures beyond routine care. Treatment decisions were made independently of study participation and reflected routine clinical practice. The NNP treatment was prescribed within its approved marketing authorization and indication. No additional procedures beyond standard care were performed for research purposes. All participants provided written informed consent, and data were anonymized prior to analysis. The study was conducted in accordance with the ethical principles of the Declaration of Helsinki.

## Results

### Socio-demographic and clinical profile of the studied population

**Fig. 1:**
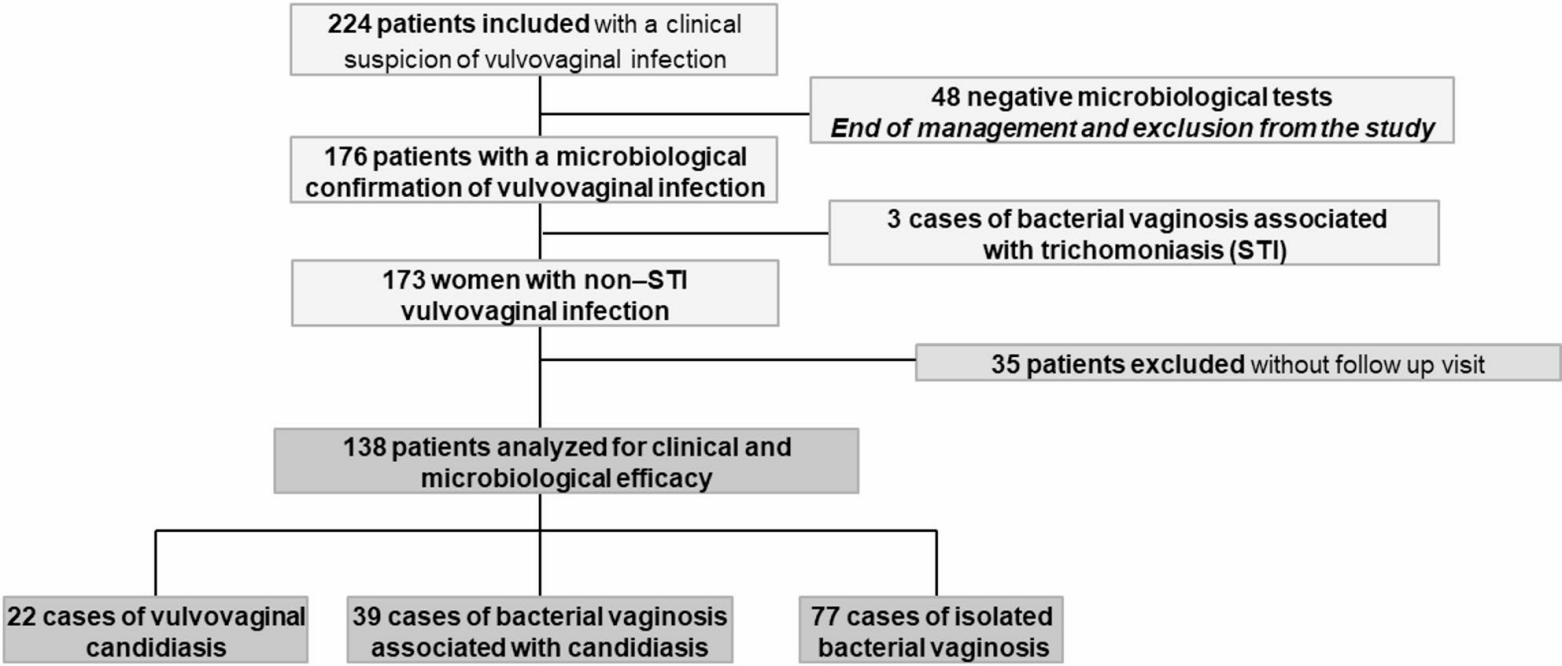
Participant flowchart

A total of 224 patients were initially enrolled in this study based on the predefined inclusion criteria (Fig. 1).

The study population was predominantly composed of women of reproductive age (15–49 years; *n* = 209/224, 93.3%), with a mean age of 32 years (ranging from 18 to 57 years). The average number of previous births was 2, and 24.2% of women had never been pregnant. Among women of reproductive age, 59.3% were in the luteal phase, 25.4% in the follicular phase, and 15.3% in the peri-ovulatory period at Day 0.

The majority of women were married (87.1%), with 75.4% living in monogamous unions. In terms of education and employment, most had completed at least primary (36.2%), secondary (34.4%) or superior (16.1%) education, and half identified as housewife (52.2%).

In terms of hygiene practices and clothing habits, a notable proportion reported vaginal antiseptic use for vaginal hygiene (36.6%), and frequent wearing of tight (59.8%) and synthetic clothing (50.1%), suggesting lifestyle factors potentially influencing vaginal health.

Most patients (76.3%) had a normal body mass index [18–30] and had no significant medical history (66.5%), though hypertension and myomectomy were each reported by 12.5% of women. A large proportion of women did not use any contraception (76.3%). A more recent infection, within the past year, was reported by 11.6% of cases (*n* = 26), most commonly identified as bacterial vaginosis (69.2%, *n* = 18/26). Previous treatments consisted mainly of antibiotics and/or antifungals administered either via topical or oral routes.

Complete socio-demographic and clinical characteristics of the participants included in this study are presented in Table 1.

**Table 1:** Characteristics of patients included in the study

Characteristics	Patients (*n* = 224)
Average age (years, mean ± SD)	32 ± 9
Marital situation [*n*(%)]	
Married	195 (87.1%)
Single	17 (7.6%)
Widow/Divorced	3 (1.3%)
Unknown	9 (4.0%)
Type of marriage [*n*(%)]	
Monogamous	147/195 (75.4%)
Polygamous	48/195 (24.6%)
Education level [*n*(%)]	
None	30 (13.4%)
Primary	81 (36.2%)
Secondary	77 (34.4%)
Higher	36 (16.1%)
Employment [*n*(%)]	
Salaried employment	45 (20.1%)
Informal self-employed	62 (27.7%)
Housewife	117 (52.2%)
Body Mass Index (BMI) [*n*(%)]	
18–30	171 (76.3%)
≥ 30	53 (23.7%)
Medical history [*n*(%)]	75 (33.5%)
Myomectomy	28 (12.5%)
Artificial skin depigmentation	10 (4.5%)
Arterial hypertension (HTA)	7 (3.1%)
Diabetes	3 (1.3%)
Sickle cell disease	1 (0.4%)
Contraceptive use [*n*(%)]	
None	171 (76.3%)
Intrauterine device (IUD)	26 (11.6%)
Combined oral contraceptive pill	14 (6.3%)
Progestin implants and injectables	13 (5.8%)
Vaginal Infection history [*n*(%)]	
< 1 year prior	26 (11.6%)
* Bacterial vaginosis*	*18 (8.0%)*
* Vaginal candidiasis*	*8 (3.6%)*

### Clinical evaluation using the WHO syndromic approach

The most common symptoms leading to the consultation were vaginal discharge (94.6%), vulvar pruritus (62.5%), pelvic pain (32.6%), dyspareunia (21.9%), vaginal burning (11.2%), and urinary symptoms (10.7%). The mean vaginal discharge abundance score was 2 (± 0.6 SD). The discharge was most often whitish (68.3%) or yellowish (21.4%), and typically had an odor described as “musty” (46.9%). The main characteristics of vaginal symptoms and leucorrhea description are summarized in Table 2.

**Table 2:** Symptoms and WHO syndromic diagnosis of the vaginal discharge among patients (*n* = 224)

Characteristics of the vaginal discharge	Number [*n*(%)]
Symptoms	
Vaginal discharge	212 (94.6%)
Vulvar pruritus	140 (62.5%)
Pelvic pain	73 (32.6%)
Dyspareunia	49 (21.9%)
Vaginal burning	25 (11.2%)
Urinary symptoms	24 (10.7%)
Color of vaginal discharge	
Whitish	153 (68.3%)
Yellowish	48 (21.4%)
Grayish	18 (8.0%)
Chocolate-colored	3 (1.3%)
Greenish	2 (0.9%)
Odor of vaginal discharge	
Musty odor	105 (46.9%)
Rotten fish	72 (32.1%)
None	47 (21.0%)
Suspected type of infection	
Candida vaginitis	79 (35.3%)
Bacterial vaginosis	105 (46.9%)
Aerobic vaginitis	22 (9.8%)
Mixed vaginitis	16 (7.1%)
Trichomoniasis	2 (0.9%)

On clinical examination, combined vaginal and abdominal palpation revealed vaginal mucosal inflammation in 27.2% of cases, cervicitis in 23.7%, and abnormal cervical mucus in 45.1%. Based on the WHO syndromic approach, the most frequently suspected infections were bacterial vaginosis (46.9%) and vaginal candidiasis (35.3%).

### Bacteriological evaluation using the microbiologic approach

Microscopic analysis of the vaginal swabs revealed an average of 8 leukocytes per field (± 5 SD). Based on lactobacillary grading, the vaginal microbiota was predominantly classified as grade IV (54.0%) or grade III (31.7%) (Table 3).

**Table 3:** Distribution of vaginal microbiota class, vaginal pH and Nugent score among patients

	Patients (*n* = 224)
Classification of vaginal microbiota [*n* (%)]	
I	5 (2.2%)
II	24 (10.7%)
III	71 (31.7%)
IV	121 (54.0%)
V	3 (1.3%)
Vaginal pH [*n* (%)]	
1–3	10 (4.5%)
4–6	170 (75.9%)
7–10	44 (19.6%)
Nugent score [*n* (%)]	
1–3	55 (24.6%)
4–6	33 (14.7%)
≥ 7	136 (60.7%)

The potassium hydroxide (KOH) test was positive in 62.1% of patients (*n* = 139), and Clue cells were identified in 55.4% of cases (*n* = 124). The average vaginal pH was 5.0 (± 1 SD), with the majority of patients (75.9%) showing a pH between 4 and 6 (Table 3).

The Amsel criteria were positive in 63.4% of patients (*n* = 142). The distribution of Nugent scores was as follows : 24.6% of patients had normal flora (Nugent score [1–3]), 14.7% had intermediate flora (Nugent score [4–6]), and 60.7% having a score ≥ 7, indicating a shift toward bacterial vaginosis (Table 3).

**Fig. 2:**
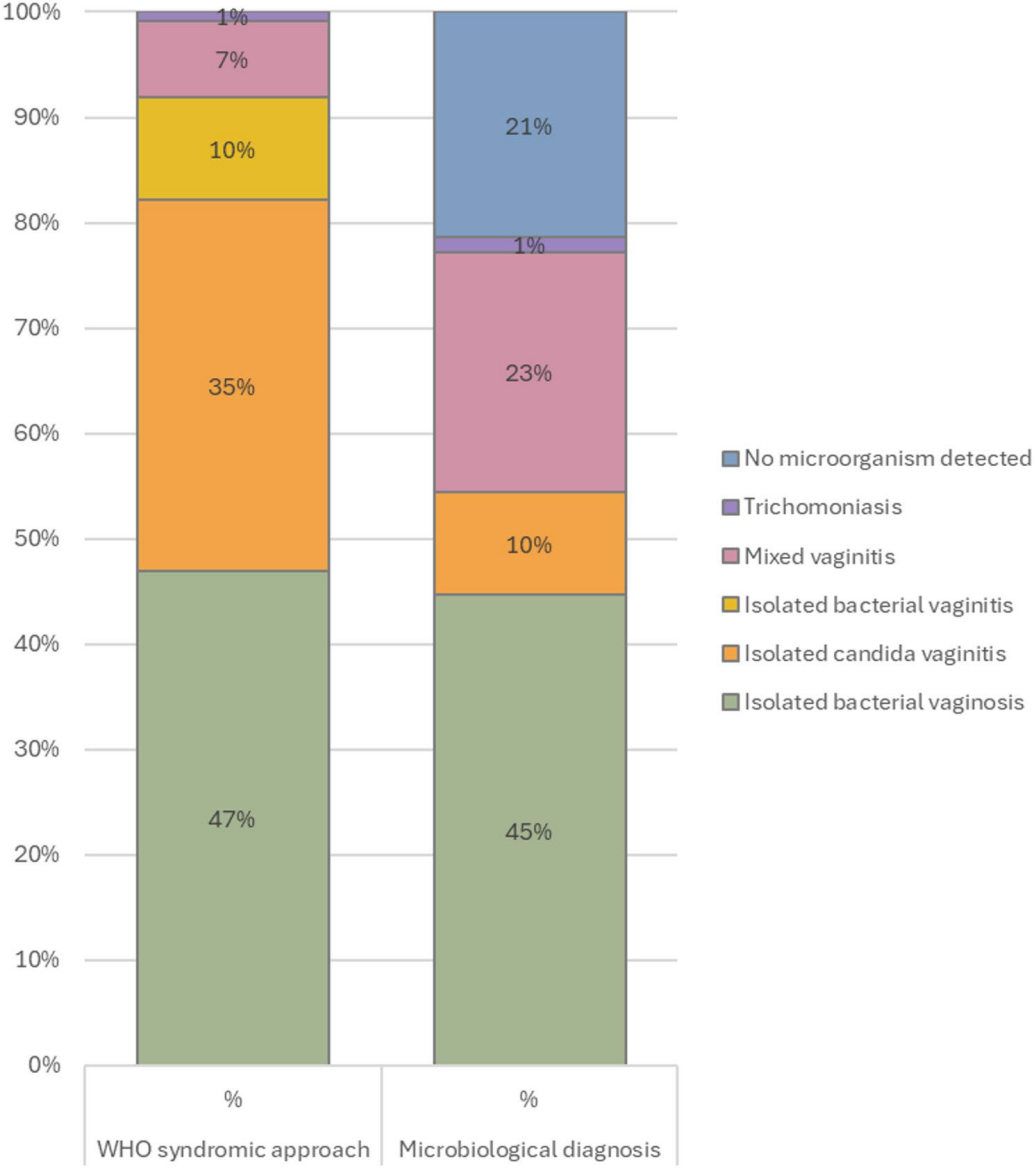
Distribution of vulvovaginal infections according to WHO syndromic and microbiological approaches (*n* = 224)

Overall, the microorganisms isolated after culture were distributed as follows: 100 cases (44.6%) of isolated bacterial vaginosis; 22 cases (9.8%) of isolated candidiasis; 51 cases (22.8%) of mixed infection caused by bacterial vaginosis associated with candidiasis; 3 cases of trichomoniasis (1.3%) associated either with bacterial vaginosis or with bacterial vaginosis and candidiasis. In 48 patients (21.4%), no microorganisms were detected. (Fig. 2).

Overall, one or more pathogens responsible for bacterial vaginosis were identified in 154 cases, representing 87.5% of all positive microbiological samples (*n* = 176 total). *Candida* species were detected in 74 patients (42.0%). The majority were *Candida albicans* alone (*n* = 54; 73%), followed by *Candida glabrata* alone (*n* = 5; 6.8%), *Candida albicans* associated with *Candida glabrata* (*n* = 14; 18.9%), and *Candida albicans* associated with *Gardnerella* and *Trichomonas vaginalis* (*n* = 1; 1.4%).

In case of bacterial vaginosis, *Gardnerella vaginalis* was identified in all of these cases, and was associated with *Mobiluncus* in 10 cases (6.5%).

### Concordance between clinical suspicion and microbiological results

Among the 224 patients with clinically suspected vulvovaginal infection, isolated bacterial vaginosis was the most commonly suspected infection (105 cases, 46.9%), and it was confirmed in 100 cases through bacteriological analysis, yielding a sensitivity of 100%, a specificity of 96.1%, and Matthews correlation coefficient of 0.96 and a Cohen’s kappa coefficient of 0.96, indicating substantial agreement.

Isolated vaginal candidiasis was clinically suspected in 79 patients but confirmed in only 22 cases, with a sensitivity of 100% and a specificity of 78.0%, and a Matthews correlation coefficient of 0.47 and a Cohen’s kappa value of 0.36, also reflecting moderate agreement and a low specificity despite very high sensitivity.

The syndromic approach has proven even less effective in predicting other types of vulvovaginal infections, particularly mixed infection, with a Matthews correlation coefficient of 0.70, and a Cohen’s kappa value of 0.41.

The summary of clinical and microbiological concordance by type of infection is presented in Table 4.

**Table 4:** Concordance between initially clinical suspected infections and microbiological results

Type of infection	Clinical suspicion (*N* = 224)	Positive bacteriological isolates (176/224)^1^	Sensitivity	Specificity	Positive Predictive Value	Negative Predictive Value	Matthews Correlation Coefficient^2^	Cohen’s Kappa test^3^
Isolated bacterial vaginosis	105 (46.9%)	100	100%	96.1%	95.2%	100%	0.96	0.96
Isolated vaginal candidiasis	79 (35.3%)	22	100%	78.0%	27.8%	100%	0.47	0.36
Bacterial vaginitis*	22 (9.8%)	0	-	-	-	-	-	-
Mixed vaginitis	16 (7.1%)	51	59.3%	100%	100%	83.1%	0.70	0.41
Trichomoniasis	2 (0.9%)	3	60%	100%	100%	99.1%	0.77	0.50

* Of note, as no cases of isolated bacterial vaginitis were confirmed by bacteriological analysis, sensitivity and specificity measurements could not be calculated

^1^224 microbiological samples analyzed, isolation of one or more organisms in 176 cases and absence of organisms in 48 cases

^2^Matthews correlation coefficient: ≥1 = perfect prediction, < 0.6 = random prediction

^3^Cohen’s Kappa index: 0.00-0.20 = very weak agreement − 0.21–0.40 = weak agreement − 0.41–0.60 = moderate agreement − 0.60–0.80 = strong agreement − 0.81-1.00 = almost perfect agreement

Statistical analysis showed that the WHO syndromic approach had good specificity and excellent sensitivity in predicting isolated bacterial vaginosis. For isolated candidiasis, the WHO syndromic approach demonstrated excellent sensitivity but low specificity. In the case of mixed vaginitis, both sensitivity and specificity were low.

### Factors associated with the occurrence of infection

The distribution of age, contraceptive use, and body mass index according to the type of vulvovaginal infection among patients diagnosed positively with one or more pathogens following bacteriological analysis (*n* = 176) is presented in Additional Table 1. Following the reception of bacteriological results, the three patients diagnosed with trichomoniasis were subjected to treatment adjustment and subsequently excluded from further analysis, as specified in the protocol (*n* = 173).

Bacterial vaginosis seems more prevalent among women aged 31–45 years (53.0%), while vaginal candidiasis was most common in the 18–30 age group (45.5%). Mixed infections were also more frequently observed among younger and middle-aged women (47.1% and 49.0%, respectively).

The majority of patients with any type of vulvovaginal infection reported not using any contraception (72.8%). Use of intrauterine devices and oral contraceptive pills was low with 13.3% and 7.5%, respectively. Other contraceptive methods included progestin-only injectables and progestin implants used by 11 patients.

Most patients (79.2%) had a normal body mass index (BMI) [18–30]. Vulvovaginal infections were most frequently observed in this BMI range, with vaginal candidiasis being the most common among these cases (95.5%), followed by bacterial vaginosis (78.0%).

### Evaluation of the clinical and microbiological efficacy of the NNP combination

All enrolled patients (*n* = 224) received empirical treatment immediately after bacteriological sampling, consisting of one NNP vaginal capsule daily for 12 consecutive days. Of note, 48 patients were excluded from the subsequent analyses due to negative bacteriological results. Additionally, 35 patients were also excluded because they did not complete the study and had no data available at the Day 15 follow-up (they were lost to follow-up), as well as 3 patients with associated trichomoniasis. Therefore, the following results include data from 138 patients, including 22 cases of isolated vulvovaginal candidiasis, 77 cases of isolated bacterial vaginosis and 39 cases of mixed infections (bacterial vaginosis + vulvovaginal candidiasis).

Among the 77 cases of isolated bacterial vaginosis, 92.2% reported a clinical remission and expressed a favorable perception of the treatment (*n* = 71). For mixed infections 89.7% of the patients reported a resolution of the symptoms (*n* = 35). Therefore, 91.4% of the 116 patients diagnosed with isolated or mixed bacterial vaginosis were clinically cured. In contrast, 10 patients (8.6%) reported persistent vaginal discharges, sometimes associated with vulvar pruritus and/or dyspareunia. Furthermore, clinical evaluation revealed that all 22 patients diagnosed with isolated candidiasis reported resolution of the symptoms, and were therefore considered clinically cured. Overall, clinical remission was reported in 128 patients (92.8%).

A satisfaction questionnaire was used to collect opinions among patients who developed bacterial vaginosis (*n* = 116) regarding their perception of the benefits of the NNP treatment compared to their initial clinical condition. Overall, the majority of patients reported satisfaction following the treatment, with 89.6% expressing good to very good satisfaction (33.6% and 56.0%, respectively).

**Fig. 3:**
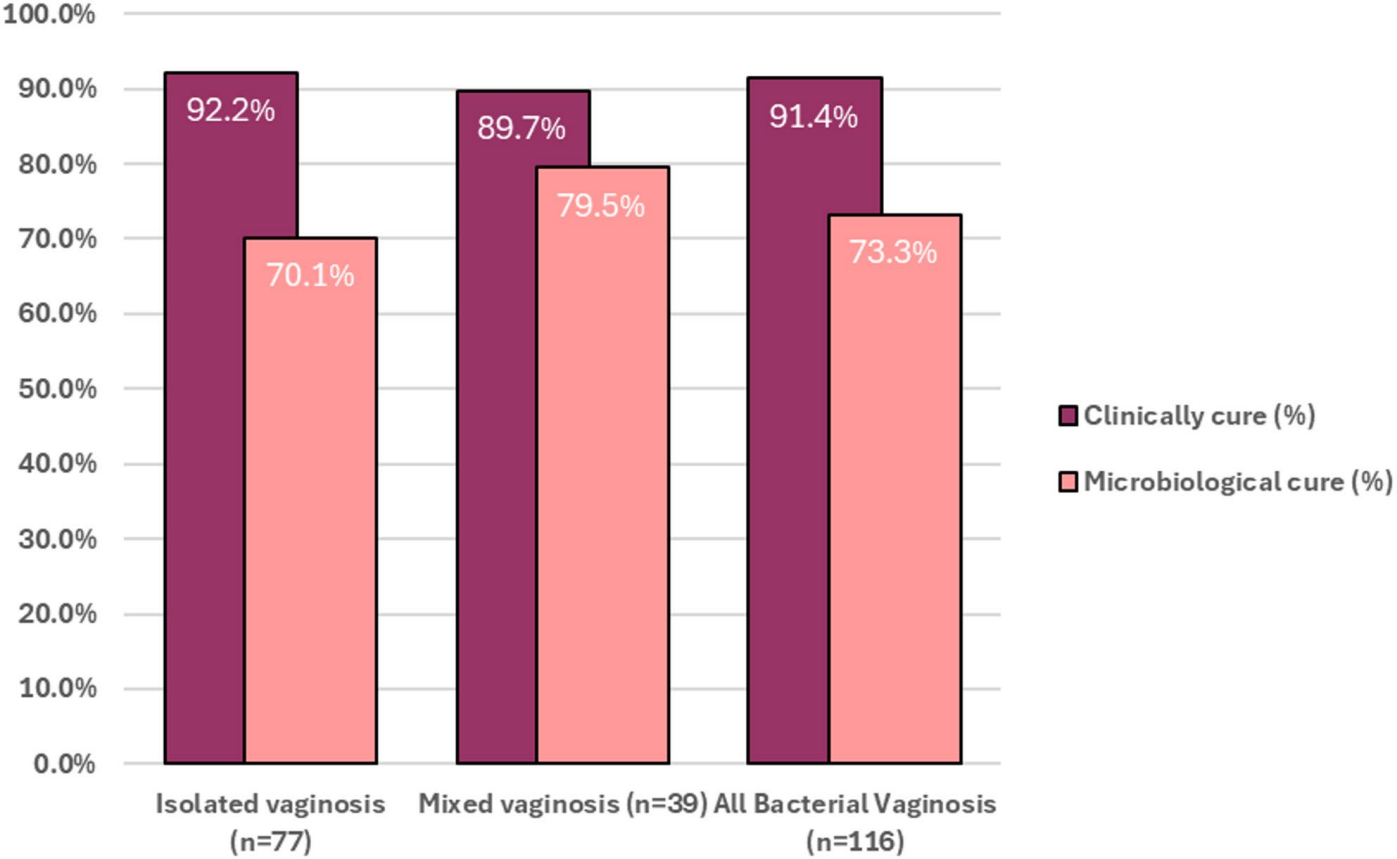
Clinical and microbiological efficacy of NNP treatment in isolated, mixed, and overall bacterial vaginosis

A bacteriological follow-up was performed at Day 15 in the 116 patients diagnosed with bacterial vaginosis. Although several microbiological parameters were assessed, including potassium hydroxide test, vaginal pH, Nugent score, Amsel criteria, and culture for pathogen identification, the primary criterion for microbiological cure was the absence of pathogens in culture, as it provides a more objective measure of treatment efficacy. In this context, microbiological cure was confirmed in 85 cases (73.3%). Specifically, among the 77 patients with isolated bacterial vaginosis, 54 achieved microbiological cure (70.1%), while 31 out of 39 patients with mixed infections were microbiologically cured (79.4%) (Fig. 3). Persistence of vaginal infection was observed in 31 patients (26.7%) based on the isolation of microorganisms. Specifically, there were 28 cases of *Gardnerella* infection, 2 mixed infections involving *Gardnerella* and *Candida*, and 1 case involving *Mobiluncus*. Among these women with positive bacteriological culture at Day 15, the Amsel criteria were not met in 2 cases, and Nugent scores were normal or intermediate in 2 and 7 patients respectively. Therefore, vaginal flora was considered intermediate or normal in 81.0% of the patient with initial diagnosis of vaginosis.

### Evaluation of the adherence and tolerance of the NNP combination

Treatment adherence, which was assessed through structured questions asked directly to patients during the follow-up visit on Day 15, was perfect in 134 patients (97.1%), while 4 patients experienced temporary discontinuation due to the onset of menstruation. Treatment tolerance was reported as excellent by 136 patients (98.6%), while two patients reported a sensation of irritation. However, it could not be determined whether this was related to the treatment itself or to the progression of the underlying infection.

## Discussion

The PETAL study aimed to better understand the epidemiological, clinical, and microbiological profile of vulvovaginal infection in Dakar, to assess the consistency between the syndromic approach with microbiological results, and finally to evaluate the clinical, microbiological efficacy and the tolerance of the Neomycin–Nystatin–Polymyxin B combination in the treatment of bacterial vaginosis.

This study revealed that the epidemiological profile of women affected by vulvovaginal infection in Dakar is commonly women in their thirties, married, with little or no formal education and without formal employment. They frequently wear tight synthetic clothing and often use antiseptic soaps for vaginal hygiene. The most common symptoms leading to the consultation were vaginal discharge and vulvar pruritus. This profile closely matches findings from earlier studies conducted in Dakar, Senegal [5], Banjul, Gambia [4], as well as more broadly across sub-Saharan Africa [6] and Ethiopia [27].

In developing countries, where establishing the etiological diagnosis of vulvovaginal infections is often challenging, the use of the WHO syndromic approach is well recognized [11]. However, opinions remain divided regarding the actual effectiveness of this method [5, 28, 29]. In our study, the most frequently suspected diagnoses were bacterial vaginosis and candidiasis. This may be explained, on the one hand, by the higher prevalence of these two conditions in African settings compared to other forms of vulvovaginal infections [4–8], and on the other hand, to the design of the WHO algorithm, which primarily relies on the assessment of vaginal discharge, a symptom more commonly observed in cases of bacterial vaginosis and candidiasis.

The effectiveness of the syndromic approach appears to be lower for detecting infections with mild or atypical clinical presentations, and mixed infections, which often alter the typical symptom profile. In our study, nearly one in four women had mixed infections, underscoring their clinical relevance and the need for diagnostic strategies capable of identifying co-infections, which are often missed when relying solely on symptom-based approaches. The limited availability of specific guidelines for mixed vulvovaginal infections further complicates accurate diagnosis and may contribute to suboptimal treatment in routine practice. Moreover, the proportion of asymptomatic cases of bacterial vaginosis can reach up to 50% [30, 31], thereby limiting the diagnostic sensitivity of clinical criteria. It should also be added that one-quarter of our patients in whom a vulvovaginal infection was suspected, and who received empirical treatment based on the syndromic approach, ultimately had negative bacteriological results. Given that microbiological cultures were examined by experienced personnel, the most likely explanation is that vulvovaginal infection symptoms may have resulted from a non-infectious process in some patients. Another explanation could be that some infectious pathogens were not able to grow, and thus to be identified according to the conventional laboratory procedures, which is the main limitation of microbiological diagnosis.

Despite these limitations, in suspected cases of bacterial vaginosis, the agreement between the syndromic approach and bacteriological examination remains highly favorable. This is supported by a sensitivity of 100%, specificity of 96%, positive predictive value of 95%, negative predictive value of 100%, and Matthews correlation coefficient and Cohen’s Kappa coefficient of 0.96 for both. Although the sensitivity of the syndromic approach is excellent for the diagnosis of candidiasis (100%), the specificity remains limited (78%), with a Matthews correlation coefficient of 0.47 and a Cohen’s Kappa coefficient of 0.36. However, these high sensitivity values should be interpreted with caution. They likely reflect the high proportion of bacterial vaginosis observed in our study population, which may have increased the probability of correct clinical identification. In addition, the single-center design may have influenced the results. Therefore, the observed diagnostic performance is specific to this context and may not be generalizable to other settings or populations. In addition, this suggests that when using the WHO syndromic approach, mixed infections may be underdiagnosed, as candidiasis tends to dominate the clinical picture and can therefore mask the presence of concurrent bacterial vaginosis.

Regarding microbiological diagnosis, there was consistency across the three key parameters used to confirm bacterial vaginosis: Amsel criteria, Nugent score, and pathogen identification, each of which contributing in establishing a definitive diagnosis [4, 5, 25, 26, 32, 33].

The most frequently isolated microorganism was *Gardnerella vaginalis*, which is consistent with the growing predominance of bacterial vaginosis among the causes of vulvovaginal infections [4–8, 12, 32, 34, 35]. This distribution appears to be more pronounced in African and Afro-descendant women [12, 34, 36] compared to Caucasian women [3, 12, 13, 34]. The high prevalence of bacterial vaginosis may be associated with several contributing factors, including low socioeconomic status, overweight or obesity, vaginal douching practices, widespread use of antibiotics, particularly azithromycin during the peri- and post-COVID period (coronavirus disease), and psychological stress [12, 13, 37, 38]. The current prevalence of modern contraceptive use among women of reproductive age in the Dakar region is estimated at 38.1% [39]. The fact that only a small proportion of patients who developed bacterial vaginosis (11%) were using contraception supports the growing reconsideration of the previously assumed association between contraceptive use, particularly copper intrauterine devices, and the occurrence of bacterial vaginosis [12, 13, 40, 41].

The increasing prevalence of bacterial vaginosis among women of reproductive age raises concerns about its growing association with pregnancy. This condition is linked to a range of obstetric complications, including miscarriage, premature rupture of membranes, preterm delivery, low birth weight, and neonatal infections [8, 31, 42]. Beyond obstetric and neonatal outcomes, bacterial vaginosis also increases a woman’s susceptibility to sexually transmitted infections, including human immunodeficiency virus (HIV), as well as chronic human papillomavirus (HPV) infection and pelvic inflammatory disease (PID), which can lead to chronic pelvic pain and impaired fertility through endometritis or tubal obstruction [12, 43]. Despite significant advances over the past 50 years, bacterial vaginosis remains a subject of ongoing controversy. Its definition is still debated by some authors that consider it a true disease, while others describe it as a form of vaginal dysbiosis. The vaginal microbiota is known to undergo significant changes, some of which may be physiological and mistakenly interpreted as pathological [12, 13]. A wide range of saprophytic bacteria are normally present in a healthy vagina, and bacterial vaginosis may occur when this natural balance is disrupted by the overgrowth of pathogenic microorganisms. The overgrowth of pathogens progressively replaces lactobacilli, but the precise threshold at which this shift becomes pathological remains unclear and widely debated [3, 12, 27, 37]. Recent advances in molecular biology have improved our understanding of the vaginal microbiome’s complexity and have helped explain the important variations observed based on geography, ethnicity, reproductive life stage, and pregnancy status [13, 37].

A significant area of debate concerns the characterization of bacterial vaginosis based on vaginal microbiota profiles, particularly Community State Type IV (CST IV), which is often associated with a Nugent score above 7 but may present without clinical symptoms. This raises the question of whether asymptomatic CST IV profiles should be classified as bacterial vaginosis. Indeed, studies have shown that some women with CST IV microbiota remain asymptomatic and are considered healthy, suggesting that microbiota composition alone is insufficient to define pathological states [44]. In our study, discrepancies were observed between positive cultures and Nugent scores at Day 15. Among the 31 patients with persistent positive cultures at Day 15, 9 presented a normal (*n* = 2) or intermediate (*n* = 7) Nugent score. While culture was used as the decisive criterion according to the study protocol, it should not be regarded as a universal gold standard, as no single reference method exists for BV diagnosis. Nugent and Amsel criteria were systematically assessed and reported, reflecting the ongoing debate regarding the most appropriate diagnostic approach. This illustrates the inherent difficulties in interpreting microbiological criteria. In populations where CST IV profiles may be common, a Nugent score above 3 does not necessarily correspond to clinically active bacterial vaginosis in the absence of symptoms. As clinical resolution remains the primary therapeutic objective, these findings support the need for a combined interpretation of clinical and microbiological parameters to provide the most objective assessment of treatment success. They also underline the limitations of conventional diagnostic tools such as Amsel and Nugent scores, whose interpretations may be influenced by microbiome composition, potentially limiting their reliability in diverse populations [45]. In light of these uncertainties, the present study used a pragmatic and objective diagnostic approach, based on pathogen identification from bacteriological cultures. However, it must be acknowledged that microbiological criteria alone may not fully reflect the clinical or biological complexity of vaginal health. More integrative models combining epidemiological, microscopic, culture-based, and sequencing techniques are needed to better define and manage conditions like vulvovaginal infections [46].

Although nitro-5-imidazoles remain the standard therapy for bacterial vaginosis, their variable efficacy and frequent adverse effects highlight the need for better-tolerated alternatives. Feuillolay et al. (2025) reported that the NNP combination exhibits rapid and strong in vitro bactericidal activity against the main pathogens responsible for bacterial vaginosis, whereas metronidazole showed limited activity after short-term contact [17]. However, since in vitro results do not guarantee clinical effectiveness, the in vivo efficacy of the NNP combination as treatment for bacterial vaginosis was evaluated in the present study. The present findings support the relevance of evaluating novel therapeutic options such as the NNP combination in real-life clinical settings. A clinical success rate of 91.4% and a microbiological cure rate of 73.3% were demonstrated. This microbiological cure rate is comparable to short-term cure rates reported for metronidazole, which typically range from 60% to 80% depending on the formulation, administration route, and follow-up duration [15, 47]. Interestingly, the treatment showed higher microbiological cure rates in mixed bacterial vaginosis cases compared to isolated forms, which is noteworthy given the complexity of co-infections. The discrepancy observed between the high clinical remission rate (91.4%) and the microbiological cure rate (73.3%) may be partly explained by asymptomatic colonization or the persistence of non-pathogenic bacterial profiles. Longer-term follow-up would be valuable to better understand the clinical relevance of residual microbiological findings. Notably, most microbiological failures in this study were associated with the persistence of *Gardnerella vaginalis*, either as a single pathogen or in mixed infections. Considering that CST IV microbiota profiles commonly include *Gardnerella vaginalis* without necessarily correlating with symptoms, such cases may reflect persistent colonization or dysbiosis rather than true clinical failure. Nevertheless, these findings suggest that the NNP combination could be an effective treatment for polymicrobial infection, in particular when *Candida* spp. and *Gardnerella spp.* are interacting. In addition, both treatment adherence and tolerance were excellent. Although comparative study will help to confirm these results, the NNP combination could represent a valuable alternative to imidazole-based treatments. It should be noted that our present study is the first to report clinical efficacy data of the NNP combination specifically in sub-Saharan women. This is particularly relevant as the vaginal microbiota and causative pathogens may differ from those in Western populations. Moreover, while previous studies evaluated NNP efficacy across various etiologies, our study is the first to suggest its effectiveness specifically in women with bacterial vaginosis.

The main limitation of this study is its observational single-arm design, where the absence of a comparator group limits the ability to draw definitive conclusions. However, the use of both clinical criteria and microbiological assessment, including culture, aims at providing a robust and objective evaluation of treatment outcomes. In addition, the relatively short follow-up period (Day 15), although consistent with routine clinical practice for assessing treatment response, does not allow evaluation of longer-term outcomes such as recurrence. One important point to consider is the potential impact of missing follow-up data. In total, 35 patients were lost to follow-up, which could represent a potential source of attrition bias. However, loss to follow-up is relatively common in real-life clinical settings, particularly when symptoms improve after treatment, and is unlikely to have substantially affected the overall efficacy estimates.

Regardless of treatment efficacy, bacterial vaginosis tends to recur over time, highlighting the importance of effective preventive strategies and some women may still present positive bacteriological results even after successful treatment and resolution of clinical symptoms. Recent evidence suggests that untreated male partners may serve as reservoirs of reinfection. In a randomized controlled trial, Vodstrcil et al. (2025) showed that concurrent treatment of male partners using oral metronidazole and topical clindamycin significantly reduced the recurrence of bacterial vaginosis within 12 weeks compared with standard care. These findings support the potential benefit of partner-based interventions to reduce recurrence [48]. Promoting appropriate hygiene practices, as well as supporting the restoration of the vaginal microbiota with probiotics [13, 49–52] could help re-establish a healthy microbial balance and therefore reduce the risk of recurrence, which remains a major challenge in managing bacterial vaginosis. Importantly, the in vitro study by Neut (2015) demonstrated that the three main Lactobacillus strains responsible for maintaining vaginal balance were not affected by the NNP combination at expected topical concentrations [53], suggesting that the NNP formulation does not disrupt the protective vaginal microbiota. By preserving beneficial flora while effectively targeting pathogenic organisms, this treatment offers a significant advantage in maintaining microbial balance and reducing the risk of recurrent infections. Future efforts should therefore focus on preserving vaginal microbiota health, particularly through appropriate genital hygiene practices and, when indicated, probiotic supplementation [54, 55].

## Conclusions

In Dakar, vulvovaginal infection is predominantly caused by bacterial vaginosis, while in one-third of cases, it involved mixed vaginal infection, with bacterial vaginosis frequently associated with candidiasis. The WHO syndromic approach remains effective in predicting the bacterial vaginosis, although its ability to detect mixed infections remains limited. The PETAL study suggests that the Neomycin, Nystatin, Polymyxin B combination could represent a clinically effective and well-tolerated treatment for managing bacterial vaginosis, particularly suited to contexts relying on syndromic diagnostic approach.

## Supplementary Information

Supplementary Material 1.

## Data Availability

The datasets used and/or analysed during the current study are available from the corresponding author on reasonable request.

## Abbreviations

BMI: Body Mass Index
COVID: Coronavirus disease
CST: Community State Type
HIV: Human Immunodeficiency Virus
HPV: Human Papillomavirus
HTA: Arterial hypertension
IUD: Intrauterine Device
KOH: Potassium hydroxide
NNP: Neomycin-Polymyxin B-Nystatin
PETAL: Polygynax Evaluation du Traitement en vie réelle au sénégAL
PID: Pelvic Inflammatory Disease
SD: Standard Deviation
WHO: World Health Organization
