# Supplementary material for: Epidemiology and management of bacterial vaginosis in Dakar, Senegal
Source: BMC Womens Health. 2026 Apr 9;26:260. doi: 10.1186/s12905-026-04443-w (PMC13217703; doi:10.1186/s12905-026-04443-w)
Supplement: Supplementary file 1 — Supplementary Material 1. [file 12905_2026_4443_MOESM1_ESM.pdf]

Additional Table 1 – Sociodemographic and clinical characteristics by type of vaginal infection among patients diagnosed positively with one or more pathogens (n=176)

Parameters	Type of infection [n(%)]					
	Bacterial vaginosis (n=100)	Vaginal candidiasis (n=22)	Mixed vaginitis (n=51)	Trichomoniasis (n=3)	**Total (n=176)**	**Chi-square (χ^2) test p-values**
**Age (years)**						
18-30	32 (32.0%)	10 (45.5%)	24 (47.1%)	2 (66.7%)	**68 (38.6%)**	**χ^2=8.738**
31-45	53 (53.0%)	8 (36.4%)	25 (49.0%)	1 (33.3%)	**87 (49.4%)**	**p=0.20**
≥ 46	15 (15.0%)	4 (18.2%)	2 (3.9%)	0 (0%)	**21 (11.9%)**	
**Contraceptive methods used**						
None	72 (72.0%)	19 (86.4%)	35 (68.6%)	2 (66.7%)	**128 (72.7%)**	
Intrauterine device (IUD)	17 (17.0%)	1 (4.5%)	5 (9.8%)	0 (0%)	**23 (13.0%)**	**χ^2=11.362**
Combined oral contraceptive pill	5 (5.0%)	1 (4.5%)	7 (13.7%)	0 (0%)	**13 (7.4%)**	**p=0.20**
Progestin implants and injectables	6 (6.0%)	1 (4.5%)	4 (7.8%)	1 (33.3%)	**12 (6.8%)**	
**Body Mass Index (BMI)**						**χ^2=4.541**
18–30	78 (78.0%)	21 (95.5%)	38 (74.5%)	2 (66.7%)	**139 (79.0%)**	**p=0.30**
>30	22 (22.0%)	1 (4.5%)	13 (25.5%)	1 (33.3%)	**37 (21.0%)**	
